# Personalized targeted therapy prescription in colorectal cancer using algorithmic analysis of RNA sequencing data

**DOI:** 10.1186/s12885-022-10177-3

**Published:** 2022-10-31

**Authors:** Maxim Sorokin, Marianna Zolotovskaia, Daniil Nikitin, Maria Suntsova, Elena Poddubskaya, Alexander Glusker, Andrew Garazha, Alexey Moisseev, Xinmin Li, Marina Sekacheva, David Naskhletashvili, Alexander Seryakov, Ye Wang, Anton Buzdin

**Affiliations:** 1 I.M. Sechenov First Moscow State Medical University, 119991 Moscow, Russia; 2 Moscow Institute of Physics and Technology, 141701 Moscow Region, Russia; 3 OmicsWay Corp, 91789 Walnut, CA USA; 4 Shemyakin-Ovchinnikov Institute of Bioorganic Chemistry, 117997 Moscow, Russia; 5 World-Class Research Center “Digital biodesign and personalized healthcare”, Sechenov First Moscow State Medical University, 119991 Moscow, Russia; 6 Clinical Center Vitamed, 121309 Moscow, Russia; 7 Department of Pathology and Laboratory Medicine, University of California, 90095 Los Angeles, CA USA; 8 N.N. Blokhin Russian Cancer Research Center, 115478 Moscow, Russia; 9 Medical Holding SM-Clinic, Moscow, Russia; 10 Core Laboratory, The Affiliated Qingdao Central Hospital of Qingdao University, Qingdao, China

**Keywords:** Transcriptomics, RNA sequencing, Molecular diagnostics, Biomarkers detection, Colorectal cancer, Targeted therapy, Oncobox, Second opinion system, Personalized medicine, Bioinformatics

## Abstract

**Background:**

Overall survival of advanced colorectal cancer (CRC) patients remains poor, and gene expression analysis could potentially complement detection of clinically relevant mutations to personalize CRC treatments.

**Methods:**

We performed RNA sequencing of formalin-fixed, paraffin-embedded (FFPE) cancer tissue samples of 23 CRC patients and interpreted the data obtained using bioinformatic method Oncobox for expression-based rating of targeted therapeutics. Oncobox ranks cancer drugs according to the efficiency score calculated using target genes expression and molecular pathway activation data. The patients had primary and metastatic CRC with metastases in liver, peritoneum, brain, adrenal gland, lymph nodes and ovary. Two patients had mutations in *NRAS*, seven others had mutated *KRAS* gene. Patients were treated by aflibercept, bevacizumab, bortezomib, cabozantinib, cetuximab, crizotinib, denosumab, panitumumab and regorafenib as monotherapy or in combination with chemotherapy, and information on the success of totally 39 lines of therapy was collected.

**Results:**

Oncobox drug efficiency score was effective biomarker that could predict treatment outcomes in the experimental cohort (AUC 0.77 for all lines of therapy and 0.91 for the first line after tumor sampling). Separately for bevacizumab, it was effective in the experimental cohort (AUC 0.87) and in 3 independent literature CRC datasets, n = 107 (AUC 0.84–0.94). It also predicted progression-free survival in univariate (Hazard ratio 0.14) and multivariate (Hazard ratio 0.066) analyses. Difference in AUC scores evidences importance of using recent biosamples for the prediction quality.

**Conclusion:**

Our results suggest that RNA sequencing analysis of tumor FFPE materials may be helpful for personalizing prescriptions of targeted therapeutics in CRC.

**Supplementary Information:**

The online version contains supplementary material available at 10.1186/s12885-022-10177-3.

## Background

Colorectal cancer (CRC) is globally the fourth most common cancer with approximately 1.8 million new cases diagnosed in 2018 [1]. CRC overall survival was increasing slowly during two last decades [2] and remains now at the level of ~ 3–5 years [3, 4] with the major factors being patient age [5] and tumor stage [6]. Several targeted cancer drugs with different molecular specificities were approved for the treatment of CRC, such as bevacizumab [7], aflibercept [8], regorafenib [9], cetuximab [10], and panitumumab [11]. New potential therapeutic molecules that act as the inhibitors of MEK, MET, RAS, RAF and PD-1/PDL-1 proteins are currently undergoing clinical trials in CRC (reviewed in [12]). Current chemotherapeutic drug prescription in CRC is generally based on the results of clinical imaging (computer tomography, magnetic resonance, positron emission tomography) and on histopathological analysis [13].

However, molecular guidance for treatment of metastatic disease was also informative and successful in several CRC subtypes. For example, activating mutations in *KRAS*, *NRAS* and *BRAF* genes are used as negative predictors for EGFR-targeted therapies [14], and microsatellite instability is regarded as an indication for immunotherapy and platinum drugs prescription [15]. In turn, in *BRAF* mutant cases combined targeting of BRAF, EGFR, and MEK by the specific therapeutics can be effective [16]. HER2-specific monoclonal antibodies and low-molecular mass tyrosine kinase inhibitors are the option to treat patients with *HER2*-amplified and wild-type *KRAS/NRAS* tumors [17, 18]. Taken together, the abovementioned genetic alterations cover approximately 50–60% of all CRC cases, leaving aside the rest 40–50% of patients [19–23]. Thus, more molecular biomarkers are needed for guiding CRC treatment, especially for the cases when the current diagnostic mutations can’t identify therapeutics or when patients don’t respond on the standardly recommended medicines/lines of treatment.

Gene expression profiles of tumor tissues can be considered as promising emerging biomarkers for cancer molecular diagnostics and personalized prescription of targeted drugs [24]. Both single gene expression profiles [25], differential gene sets/diagnostic signatures [26], and molecular pathway activation levels [27] may be useful to predict treatment outcomes for the individual tumors with regard to any associated cancer drug or treatment regimen. RNA sequencing is currently recognized as the method of choice for high throughput assessment of gene expression [28]. Further comparison of cancer gene expression levels with the normal tissues is important for delineating specific mechanisms of cancer onset, progression and responsiveness to clinical interventions [29, 30].

However, RNA sequencing profiles can be obtained using different equipment, reagents and protocols and sometimes can be poorly compatible thus making their direct comparison problematic [31, 32]. In order to obtain comparable results, ideally the same experimental platform should be used for all biosamples in a given study [32]. We recently published a collection of RNA sequencing profiles of human healthy tissues called Atlas of Normal Tissue Expression (ANTE) [33]. It contains 159 tissue samples of human healthy donors and provides relevant reference groups for twenty human organs including colon.

Finding appropriate normal expression profiles is crucial for the molecular pathway activity analysis and for modeling tumor-specific drug efficacy. For example, a bioinformatic method Oncobox calculates “balanced efficiency score” (BES) for cancer therapeutics based on a parallel analysis of drug target gene expressions and target molecular pathway activation levels in each individual tumor [34–37]. As the output, it returns personalized rating of targeted drugs [38]. This approach was demonstrated to be effective in an ongoing prospective clinical investigation [39] and also for prescription of experimental therapies in advanced cholangiocarcinoma, lung cancer, granulosa cell ovarian cancer, and gastric cancer cases [35, 40–42]. As investigated on a retrospective cohort of gastric cancer patients, it allowed robust prediction of the efficacy of ramucirumab, a vascular endothelial growth factor (VEGF) receptor-specific therapeutic monoclonal antibody [43]. However, the Oncobox method performance so far hasn’t been tested for predicting targeted drug efficiencies in the CRC patient cohorts.

In this paper, we report original clinically annotated RNA sequencing profiles for 23 CRC tumors for which we used Oncobox platform to predict individual treatment outcomes based on RNA sequencing data and compared these predictions with the real tumor response records.

## Materials and methods

### Tissue samples

All experimental biosamples were formalin fixed, paraffin embedded (FFPE) tumor tissue blocks. Prior further analyses, all biosamples were evaluated by a pathologist to confirm the tumor tissue origin and only the specimens with the content of tumor cells greater than 50% were further investigated. Samples were obtained from various medical institutions of Russia and Lithuania (Supplementary file S1). The patients were 5 men and 18 women (range 37–72 years). 13 patients had sigmoid colon cancer, 1 – cecum cancer, 1 – rectal cancer, 8 – colon cancer (nos). The samples were clinically annotated with the information about the patient’s diagnosis and clinical history. For all the biosamples, informed written consents to participate in this study (including publication of anonymized clinical, transcriptomic, and genetic data) were collected from the patients or their legal representatives. The consent procedure and the design of the study were approved by the ethical committee of Vitamed Clinic, Moscow, protocol date 16.10.17. Tumor responses to each line of therapy were characterized according to RECIST criteria for CRC [44]. For those 39 lines of therapy, the following treatment outcomes were collected. *Control over disease* was registered for 12 cases (8 *stable disease* and 4 *partial response* outcomes), and 27 therapies resulted in *progression of the disease*.

For the above 39 lines collected, targeted drugs were used alone or in combination with other chemotherapeutic schemes. The following targeted drugs were administered during these treatments: bevacizumab (n = 23), cetuximab (n = 3), panitumumab (n = 2), regorafenib (n = 3), crizotinib (n = 2), cabozantinib (n = 2), denosumab (n = 1), bortezomib (n = 1), and aflibercept (n = 2) (Table S1).

### Preparation of libraries and RNA sequencing

To isolate RNA, 10 µM - thick paraffin slices were trimmed from each FFPE tissue block using microtome. RNA was extracted from FFPE slices using QIAGEN RNeasy FFPE Kit following the manufacturer’s protocol. RNA 6000 Nano or Qubit RNA Assay kits were used to measure RNA concentration. RNA Integrity Number (RIN) was measured using Agilent 2100 bio-Analyzer. For depletion of ribosomal RNA and library construction, KAPA RNA Hyper with rRNA erase kit (HMR only) was used. Different adaptors were used for multiplexing samples in one sequencing run. Library concentrations and quality were measured using Qubit ds DNA HS Assay kit (Life Technologies) and Agilent Tapestation (Agilent). RNA sequencing was done at Department of Pathology and Laboratory Medicine, University of California Los Angeles, using Illumina HiSeq 3000 equipment for single-end sequencing, 50 bp read length, for approximately 30 million (mln) raw reads per sample). Data quality check was done on Illumina SAV. De-multiplexing was performed with Illumina Bcl2fastq2 v 2.17 program. Sequencing data were deposited in NCBI Sequencing Read Archive (SRA) under accession ID PRJNA663280.

### Processing of RNA sequencing data

RNA sequencing FASTQ files were processed with STAR aligner [45] in “GeneCounts” mode with the Ensembl human transcriptome annotation (Build version GRCh38 and transcript annotation GRCh38.89). Ensembl gene IDs were converted to HGNC gene symbols using Complete HGNC dataset (https://www.genenames.org/, database version from 2017 to 13). Totally, expression levels were established for 36 596 annotated genes with the corresponding HGNC identifiers. Statistics concerning mapping quality and reads number is stored in Supplementary file S2.

### Publicly available gene expression datasets

The following publicly available gene expression CRC datasets were used: TCGA, GSE19862, GSE19860, GSE104645, Syn2623706. TCGA cohort contains 17 patients treated with the first-line targeted drugs (bevacizumab, cetuximab, panitumumab, regorafenib). 10 patients demonstrated progressive disease, 1 – stable disease, 4 – partial response, and 2 – complete response. PFS information was available for 117 patients. GSE19860 dataset contains expression data for 12 tumor samples from patients with metastatic or recurrent CRC who were treated with bevacizumab in combination with chemotherapy (five treatment responders and seven non-responders). GSE19862 dataset contains expression profiles for seven responders and seven non-responders to bevacizumab therapy. All patients had metastatic or recurrent CRC and received bevacizumab therapy as the first- or second-line treatment. GSE104645 dataset has 193 gene expression profiles of patients with metastatic CRC, 183 of them were “pre-treatment” samples. The first-line bevacizumab treatment outcome is available for 81 patients (75 responders, 6 non-responders). Also, 80 patients with wild type status of *KRAS* gene (*KRAS*^wt^) were annotated by response to “second-line” cetuximab (54 responders, 26 non-responders). PFS data were available for 85 patients, who received bevacizumab, and for 83 patients who received cetuximab. We also used 768 gene expression profiles from Syn2623706 dataset annotated with PFS data.

### Molecular pathway analysis

Pathway activation levels were established using Oncobox analytic software [34] for 1682 molecular pathways extracted from the extracted from the public databases Reactome [46], NCI Pathway Interaction Database [47], Kyoto Encyclopedia of Genes and Genomes [48], HumanCyc [49], Biocarta [50] and Qiagen Pathway Central (available at https://www.qiagen.com/us/shop/genes-and-pathways/pathway-central/). The molecular pathways were visualized using Oncobox pathway visualization/reconstruction tool [34, 36].

The pathway activation level (PAL) scores were calculated according to Oncobox method [37]. Tumor gene expression profiles were normalized on the geometric mean tissue expression profile of the control groups to calculate PAL for each individual sample investigated^33^. PAL scores were calculated as follows

$$PA{L_p} = \sum\limits_n {AR{R_{np}}} *{\rm{lg}}\left( {CN{R_n}} \right)/\sum\limits_n {|AR{R_{np}}} |,$$

where *PAL*_*p*_ is *PAL* for pathway p, *CNR*_*n*_ is *case-to-normal ratio*, the ratio of gene *n* expression level in a tumor sample under study to an average level for the control group; *ARR* (*activator/repressor role*) is Boolean flag that depends on function of gene *n* product in pathway *p*. ARR is − 1 if gene product *n* inhibits pathway *p*; 1 if *n* activates pathway; 0 if *n* has ambiguous or unclear role in a pathway; 0.5 or − 0.5, if *n* is rather activator of a pathway or its inhibitor, respectively.

### The random permutation test

To test whether a given number of common differential genes or pathways between the three intersecting datasets is significant, 10 000 random intersections were performed. In every case, three random samples from three corresponding gene/pathway sets of the respective datasets were taken. Then these random samples were intersected for each iteration and 10 000 random sets of common genes/pathways were obtained. P-value of intersection significance was calculated as a fraction of random sets with equal or higher number of common genes/pathways than in the experimental observations.

### Ranking targeted drugs and survival analysis

Ranking of target cancer drugs using Balanced Efficiency Scores (BES) was performed as described previously [27, 38, 40]. The Oncobox software returned personalized list of target drugs in descent order of predicted efficacy. The observed clinical responses were used for validation of Oncobox predictions using ROC AUC analysis. ROC AUC was calculated using R ROCR package. Patient survival analysis and plotting were performed using R packages *survival*, *survminer* and *ggplot2*. *P*-value threshold was set to 0.05.

## Results

### Clinical data

We enrolled twenty three retrospective patients with diagnosed primary or metastatic CRC who received at least one line of therapy with targeted cancer drugs, and to whom clinical response data were available. There were eighteen female and five male 37–72 years old patients enrolled (mean age 46 y.o.). Among them eighteen had distant metastases at the time of the enrollment (Table 1; Supplementary file S1). In total, results of 39 different lines of therapy were collected, on the average 2 lines per each patient enrolled (Supplementary file S1). With relation to the date of obtaining tumor biopsy, among them 23 represented first line of chemotherapy.

**Table 1:** Outline of patient clinical and molecular information for the first line targeted therapies

ID	Sample	Targeted drug	Non-target therapy	Response	PFS ^*^	Rank ^**^	BES ^***^	T	N	Distant metastases	*RAS* ^****^
CC-1	Primary colorectal cancer	regorafenib		progressive disease	3	160	-62.1	NA	NA	NA	Mut
CC-2	Primary colorectal cancer	bevacizumab	FOLFOX	progressive disease	3	104	-0.9	3	2	Yes	Mut
CC-4	Primary colorectal cancer	panitumumab	FOLFIRI	progressive disease	2	139	-9.5	4	2	No	WT
CC-5	Primary colorectal cancer	bevacizumab	FOLFIRI	progressive disease	5	81	-0.8	NA	2	Yes	Mut
CC-6	Primary colorectal cancer	cetuximab	FOLFIRI	progressive disease	3	90	-2.6	4	2	Yes	WT
CC-18	Primary colorectal cancer	cetuximab	XELOX	progressive disease	8	137	-9.3	3	1	Yes	WT
CC-34	Primary colorectal cancer	bevacizumab	FOLFOX	progressive disease	4	19	4.4	4	2	Yes	Mut
CC-35	Liver metastasis	bevacizumab	FOLFIRI	partial response	12	54	0.1	3	1	Yes	WT
CC-41	Liver metastasis	bevacizumab	FOLFOX	stable disease	9	5	7.9	4	0	No	WT
CC-44	Primary colorectal cancer	bevacizumab	FOLFOX	stable disease	11	2	23.5	3	0	Yes	WT
CC-45	Peritoneal metastasis	bevacizumab	FOLFIRINOX	stable disease	20	13	5.1	4	1	Yes	Mut
CC-64	Brain metastasis	bevacizumab	FOLFIRI	progressive disease	5	6	24.3	3	0	Yes	WT
CC-65	Primary colorectal cancer	bevacizumab	FOLFIRINOX	partial response	6	22	3.1	4	2	Yes	Mut
CC-72	Liver metastasis	aflibercept	FOLFIRI	progressive disease	3	166	-107.9	3	2	Yes	Mut
CC_91	Small Intestine metastasis	bevacizumab	capecitabine	stable disease	13	6	9.0	3	1	Yes	Mut
CC-94	Adrenal Gland metastasis	bevacizumab	FOLFIRI	partial response	5	27	6.6	3	1	Yes	WT
CC-95	Liver metastasis	bevacizumab	FOLFOX	progressive disease	4	99	0.3	3	1	Yes	WT
CC-105	Primary colorectal cancer	regorafenib		progressive disease	3	113	-3.0	3	1	Yes	WT
CC-107	Primary colorectal cancer	bevacizumab	FOLFOX	stable disease	4	22	1.7	3	2	Yes	Mut
CC-109	Peritoneal metastasis	bevacizumab	FOLFIRI	stable disease	8	1	15.7	4	0	Yes	WT
CC-111	Peritoneal metastasis	bevacizumab	FOLFOX	progressive disease	2	165	-18.3	3	0	Yes	Mut

^*^ Progression-free survival

^**^ Rank of Oncobox Balanced Efficiency Score for a given targeted drug in a patient

^***^ Balanced Efficiency Score of a drug

^****^ Presence of known activating mutations in *KRAS* or *HRAS*

### RNA sequencing results

The CRC tissue specimens were the formalin fixed paraffin embedded (FFPE) tissue blocks stored in clinical diagnostic laboratory for 4–49 months before extraction of RNA. RNA sequencing resulted in fourteen sets of 29–41 million sequencing reads, on the average ~ 35 million reads per library. Among them, there were ~ 6–15 million reads uniquely mapped on known human Ensembl genes, on the average ~ 10 million gene-mapped reads per library. All the CRC gene expression profiles obtained here met the quality control criterion for the RNA sequencing protocol used of returning at least 2.5 million uniquely gene-mapped reads per successful library [33].

To assess data reproducibility, we added a technical control of one CRC sample (CC-9 sample) that was RNA-sequenced in four replicates using independent RNA extraction and library preparation procedures.

The gene expression profiles were then analyzed to assess if the profiles obtained are congruent with the biological nature of the biosamples under study. To this end we used principal component analysis (PCA) plot and hierarchical clustering dendrogram to visualize distributions of the experimental CRC profiles obtained here with the profiles of healthy human colon and liver tissues from ANTE database previously obtained with the same equipment and protocol [33], Fig. S1. We observed especially tight clustering for the CC-9 replicates with mean Spearman’s correlation coefficient 0.965 (Fig. S1B). The experimental CRC samples formed clear-cut clusters that were most closely located with the normal colon profiles, whereas the liver samples formed outgroup clusters (Fig. S1A, C). These results suggest that the experimental RNA sequencing profiles obtained were technically reproducible and reflected biological properties of the respective biosamples (Fig. S1). We also calculated BES for each of these four replicates to assess their reproducibility for all targeted drugs. Mean Spearman correlation coefficient was 0.926 for comparison of BES among the replicates, standard deviation values are shown in Supplementary table S2.

### Genes and molecular pathways linked with CRC treatment prognosis

We performed a search for novel gene expression based prognostic biomarkers. First, we analyzed differential gene expression in order to identify individual genes correlated with poor prognosis in colorectal cancer patients. We analyzed separately three datasets: the experimental dataset and two external datasets for validation: colorectal TCGA [51] and Syn2623706 [52]. Patients in the experimental cohort had lower PFS time, when compared to TCGA and Syn2623706. The latter may be due to increased proportion of the advanced cancers: the experimental cohort had 69% stage IV patients, in contrast to only 14.3% and 3.8% in the TCGA and Syn2623706 cohorts, respectively. For each patient we took progression-free survival (PFS) time in months and performed Cox proportional hazards survival analysis [53]. For each gene we separated patients in two cohorts: patients with DESeq2 normalized gene expression higher than median in all patients and patients with gene expression lower than median, and compared PFS time between these two groups in Cox model. For each gene we calculated log-rank p-value and Hazard Ratio that indicates relative impact of gene in overall risk of recurrency [54]. We extracted 1787, 1352 and 2770 differentially expressed genes for experimental, TCGA and Syn2623706 datasets, respectively (Supplementary table S3). However, these gene lists showed random intersection pattern (*p* = 0.38).

To further investigate molecular processes linked with the CRC prognosis, we then performed molecular pathway activation analysis using the Oncobox software [55].

In all three datasets (experimental, TCGA, and Syn2623706), we performed Cox survival analysis for each molecular pathway in the same way as for the individual gene products. Patients were stratified into two groups according to PAL value (less and higher than median PAL). We obtained log-rank test p-value < 0.05 for 83 pathways in the experimental dataset, 110 pathways in TCGA dataset, and 687 pathways in the Syn2623706 dataset. Five pathways were common for all three datasets (*p* = 0.0311 according to random permutation test, see Methods for details, Tables 2, Supplementary file S4). *KEGG Bladder cancer pathway*, *EGG cGMP PKG signaling pathway, NCI E cadherin signaling in keratinocytes pathway*, and *KEGG Aldosterone regulated sodium reabsorption pathway* were associated with unfavorable prognosis, whereas *NCI Signaling events mediated by HDAC Class II Pathway* was linked with longer PFS. Tumor stage may have an impact on PFS, therefore we also performed multivariate Cox analysis, which included stage as a variable. All the above pathways remained significant also in the multivariate analysis, except for *KEGG Aldosterone regulated sodium reabsorption pathway* (Supplementary table S4)”.

**Table 2:** *P*-values of log-rank test and Hazard ratio for pathways, which were associated with PFS in three datasets investigated (experimental, TCGA, and Syn2623706)

Dataset	experimental	TCGA	Syn2623706	experimental	TCGA	Syn2623706
Pathway	Hazard ratio			p-value of log-rank test		
*KEGG Bladder cancer Pathway*	6.21	1.47	0.72	1.84E-02	4.80E-02	1.84E-02
*NCI Signaling events mediated by HDAC Class II Pathway*	0.16	0.63	0.56	6.11E-05	1.68E-02	6.11E-05
*KEGG cGMP PKG signaling Pathway*	6.21	1.46	1.56	1.54E-03	4.30E-02	1.54E-03
*NCI E cadherin signaling in keratinocytes Pathway*	6.21	1.71	1.56	1.64E-03	5.92E-03	1.64E-03
*KEGG Aldosterone regulated sodium reabsorption Pathway*	5.38	1.50	1.54	2.52E-03	3.11E-02	2.52E-03

**Fig. 1:**
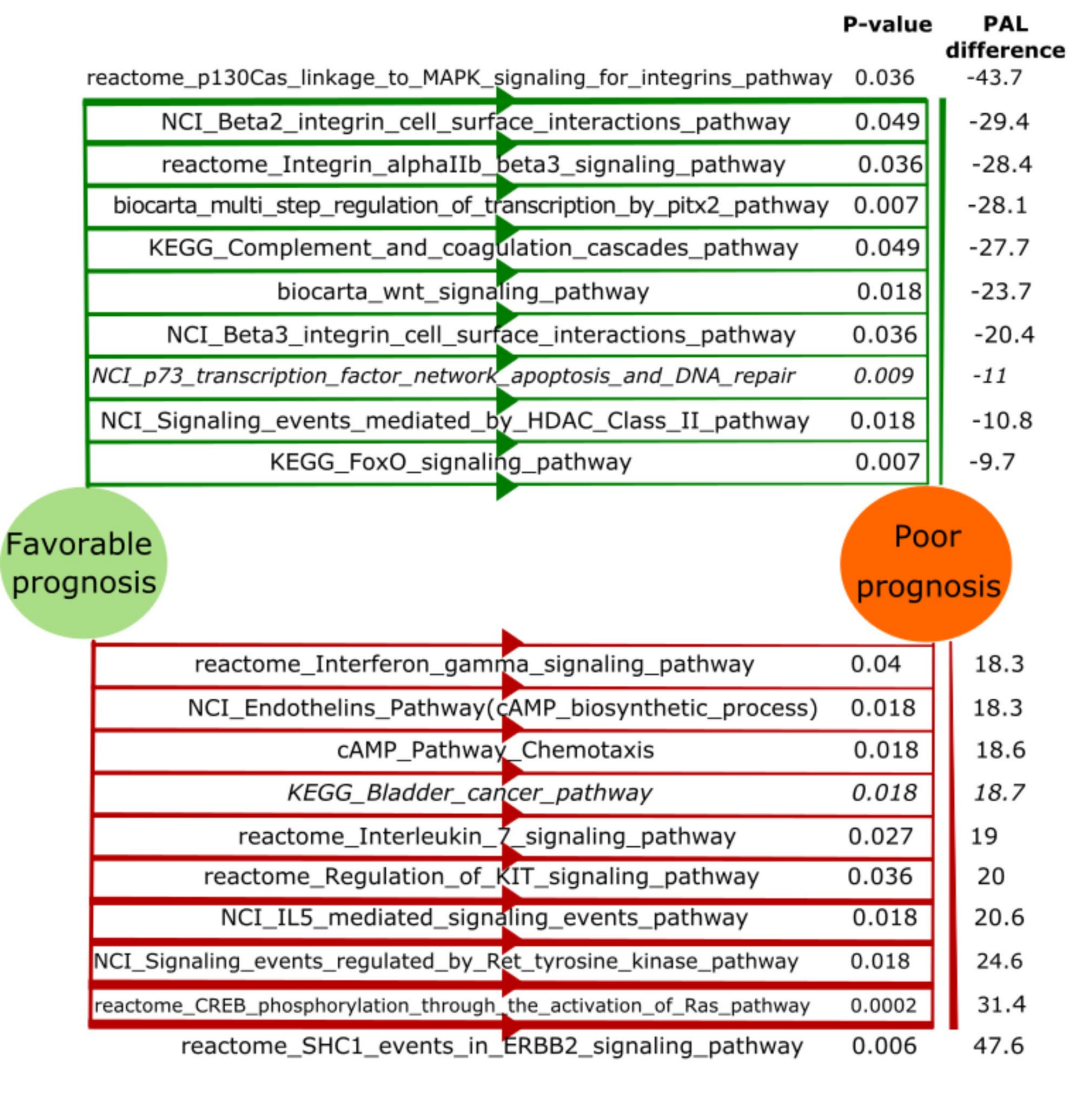
Top 10 upregulated and inhibited pathways sorted by PAL values for comparison between poor and favorable prognosis CRC samples. Pathways upregulated in favorable prognosis group are shown on the top, pathways upregulated in poor prognosis group shown on the bottom. Pathways validated using TCGA dataset are shown in italic

**Fig. 2:**
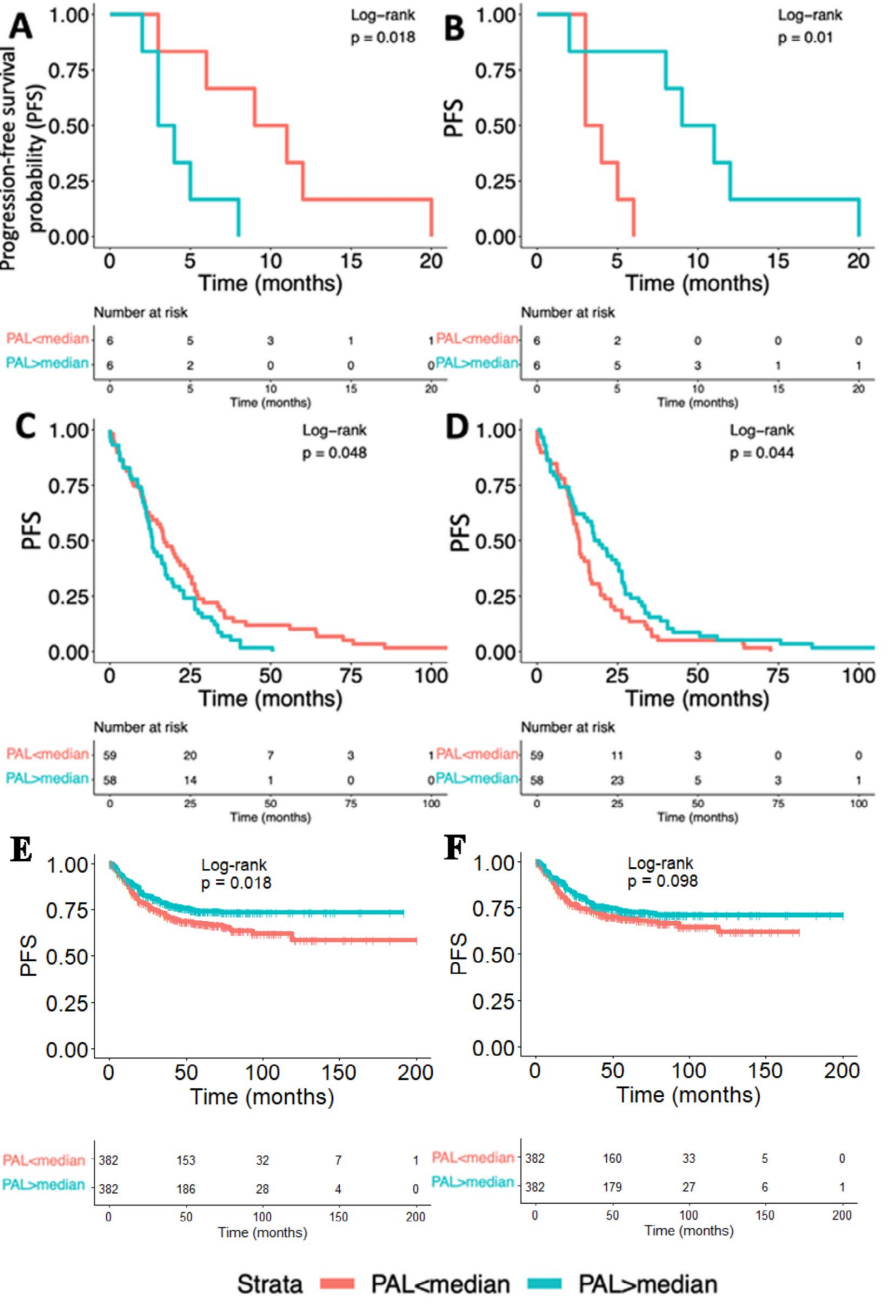
Kaplan-Meier plots and risk tables for the validated pathways. **(a) –** experimental samples, *KEGG Bladder cancer Main Pathway*. **(b)** - experimental samples, *NCI p73 transcription factor network Pathway apoptosis and DNA repair*. **(c) –** TCGA samples, *KEGG Bladder cancer Main Pathway*. **(d)** – TCGA samples, *NCI p73 transcription factor network Pathway apoptosis and DNA repair.***(e) –** Syn2623706 samples, *KEGG Bladder cancer Main Pathway*. **(e)** – Syn2623706 samples, *NCI p73 transcription factor network Pathway apoptosis and DNA repair*

We separately analyzed top 10 pathways associated with poor and favorable prognosis in the experimental dataset. For that we calculated the difference between mean PAL in patients with poor prognosis and mean PAL in patients with favorable prognosis (Supplementary table S4). The pathways with the highest/lowest PAL difference are shown on Fig. 1. The most strongly downregulated pathways in experimental patients with favorable prognosis were “*reactome SHC1 events in ERBB2 signaling Main Pathway”, “reactome CREB phosphorylation through the activation of Ras Main Pathway”, “NCI Signaling events regulated by Ret tyrosine kinase Main Pathway”, “NCI IL5 mediated signaling events Main Pathway”, “reactome Regulation of KIT signaling Main Pathway”, “reactome Interleukin 7 signaling Main Pathway”, “KEGG Bladder cancer Main Pathway”, “cAMP Pathway Chemotaxis”, “NCI Endothelins Pathway (cAMP biosynthetic process)”, “reactome Interferon gamma signaling Main Pathway”. KEGG Bladder cancer pathway* was associated with poor prognosis in two datasets (experimental and TCGA), but was associated with favorable prognosis in the Syn2623706 dataset. However, Syn2623706 dataset contains data for only 5793 genes, and only two of them are connected to *KEGG Bladder cancer pathway*, thus the corresponding PAL values for Syn2623706 could be irrelevant. Kaplan-Meier plots for *KEGG Bladder cancer Main Pathway* are shown on Fig. 2.

The most strongly upregulated pathways in experimental patients with favorable prognosis were “reactome p130Cas linkage to MAPK signaling for integrins Main Pathway”, “NCI Beta2 integrin cell surface interactions Main Pathway”, “reactome Integrin alphaIIb beta3 signaling Main Pathway”, “biocarta multi step regulation of transcription by pitx2 Main Pathway”, “KEGG Complement and coagulation cascades Main Pathway”, “biocarta wnt signaling Main Pathway”, “NCI Beta3 integrin cell surface interactions Main Pathway”, “NCI p73 transcription factor network Pathway apoptosis and DNA repair”, “NCI Signaling events mediated by HDAC Class II Main Pathway”, “KEGG FoxO signaling Main Pathway”. NCI p73 transcription factor network Pathway apoptosis and DNA repair was the pathway with the highest PAL difference between patients with poor and favorable prognosis in the experimental dataset, which was also significant in TCGA dataset (Figs. 1 and 2). The same trend was observed also in the Syn2623706 dataset; however, this difference did not reach statistical significance (p = 0.098).

**Fig. 3:**
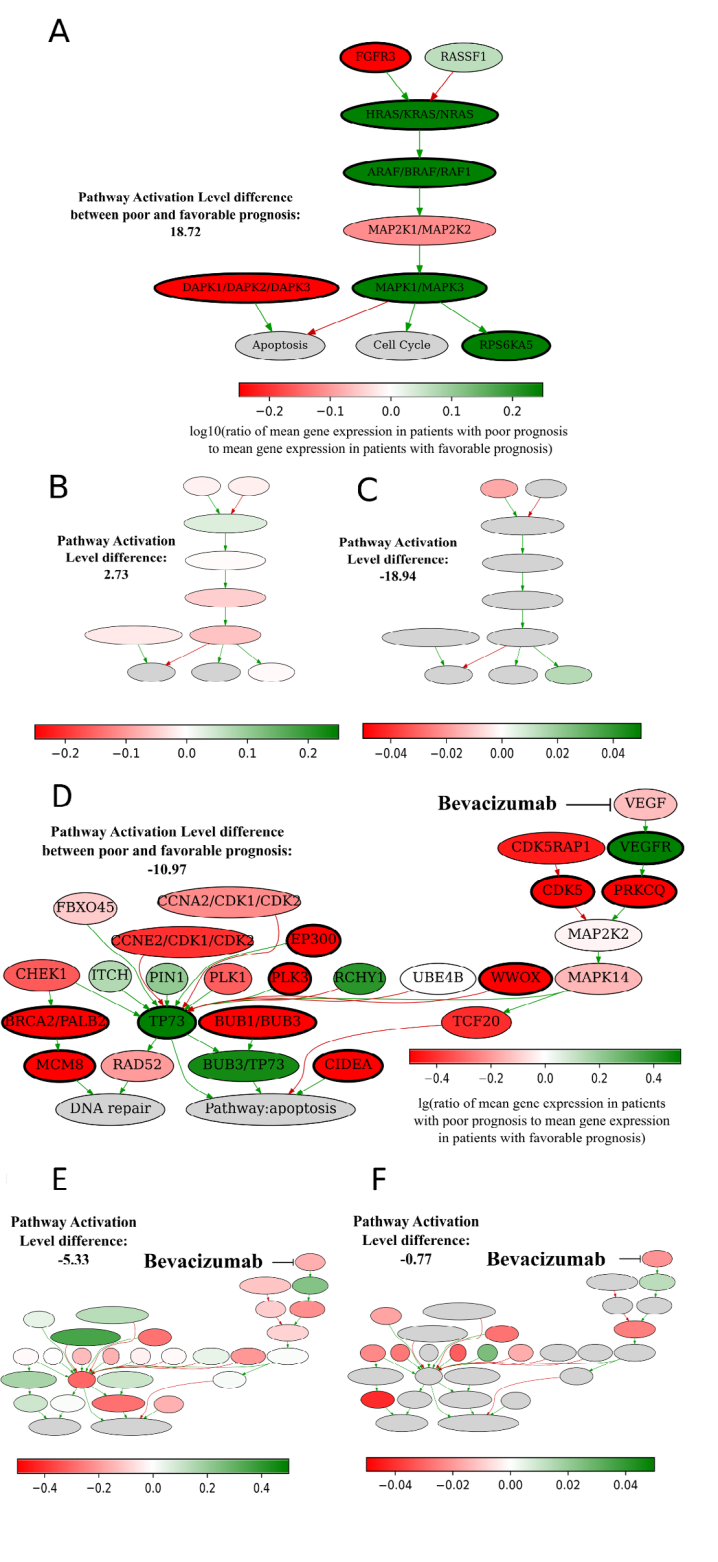
Activation schemes of *KEGG Bladder cancer Main Pathway* (**a-c**) and *NCI p73 transcription factor network Pathway apoptosis and DNA repair* (**d-f**) in experimental (**ad**), TCGA (**be**) and Syn2623706 (cf.) CRC samples. Color indicates log10-transformed ratio of mean gene expression values in patients with poor prognosis normalized on gene expression in patients with favorable prognosis. Bold margins indicate most strongly up/downregulated nodes. Patients were divided into poor and favorable prognosis groups by median survival for experimental and TCGA datasets. Because median survival was undefined for Syn2623706 dataset, poor and favorable prognosis was determined relatively median of survival time for non-censored patients

The pathway activation schemes for *NCI p73 transcription factor network Pathway apoptosis and DNA repair, KEGG Bladder cancer Pathway* were visualized and shown on Fig. 3. The *KEGG bladder cancer pathway* activation in poor prognosis CRCs was mostly due to increased expression of functional nodes HRAS/KRAS/NRAS, ARAF/BRAF/RAF1, MAPK1/MAPK3, and RPS6KA5 (Fig. 3 A-C). Interestingly, this pathway that was upregulated in poor-prognosis cancers lacks known molecular targets for drugs investigated in this study (Table 3).

**Table 3:** Characteristic of targeted drugs investigated in the experimental group

Drug generic name	Molecular target(s)	Reference for molecular target	Drug use in CRC	Reference for drug use
Bevacizumab	VEGF	[56]	Approved for advanced CRC	[7]
Aflibercept	VEGFA, VEGFB, PGF	[57]	Approved for CRC	[58]
Bortezomib	PSMB5, PSMB1	[59]	Off-label for CRC	[60]
Cabozantinib	MET, KDR	[61]	Off-label for CRC	[62]
Cetuximab	EGFR	[63] [64]	Approved for metastatic CRC	[65]
Crizotinib	ALK, MET	[66]	Off-label for CRC	[67]
Panitumumab	EGFR	[68]	Approved for metastatic CRC	[69]
Regorafenib	RET, VEGFR1, VEGFR2, VEGFR3, KIT, PDGFR-alpha, PDGFR-beta, FGFR1, FGFR2, TIE2, DDR2, TrkA, Eph2A, RAF-1, BRAF, SAPK2, PTK5, and Abl	[70]	Approved for metastatic CRC	[9]
Denosumab	TNFSF11	[71]	Off-label for CRC	[72]

The *NCI p73 transcription factor network Pathway apoptosis and DNA repair pathway* was, in contrast, enhanced in favorable prognosis patients in three datasets, but showed borderline significance for Syn2623706 (Fig. 2). On its pathway activation scheme, we observed that this pathway was activated in favorable prognosis group mostly due to increased expression of nodes TP73, BUB1/BUB3, BRCA2/PALB2, MCM8, CIDEA, WWOX, EP300, PLK3, CDK5 and PRKCQ (Fig. 3DE). In turn, this pathway contains VEGF - molecular target of bevacizumab, one of most common CRC drugs (Table 3), which was not among the top activated nodes in favorable prognosis CRCs still was upregulated in them in three datasets (Fig. 3D-F). This favorable prognosis association in the experimental dataset is in line with the clinical records that 15 experimental CRC patients received bevacizumab during the first line of treatment.

### Validation of algorithmic prediction of drug response for CRC patients

Oncobox platform is unique in its possibility of algorithmic individual prioritizing of targeted cancer drugs using high-throughput gene expression data and pathway activation profiles [24, 27, 38]. The therapeutics are ranked according to their simulated abilities to inhibit aberrantly regulated molecular pathways and drug target genes [38]. We used this platform to build personalized rating of targeted drugs for each individual CRC sample under investigation. To this end, the RNA sequencing profiles obtained for the experimental CRC samples were individually compared with the set of six healthy colon tissue profiles from the ANTE collection [33]. We chose this type of normalization instead of other collections available from TCGA [51] and GTEX [73] project databases because of identical equipment and sequencing protocols. This was also reflected by closer PCA clustering pattern of experimental CRC profiles and ANTE norms compared to TCGA and GTEX norms (Supplementary Figure S1A).

**Fig. 4:**
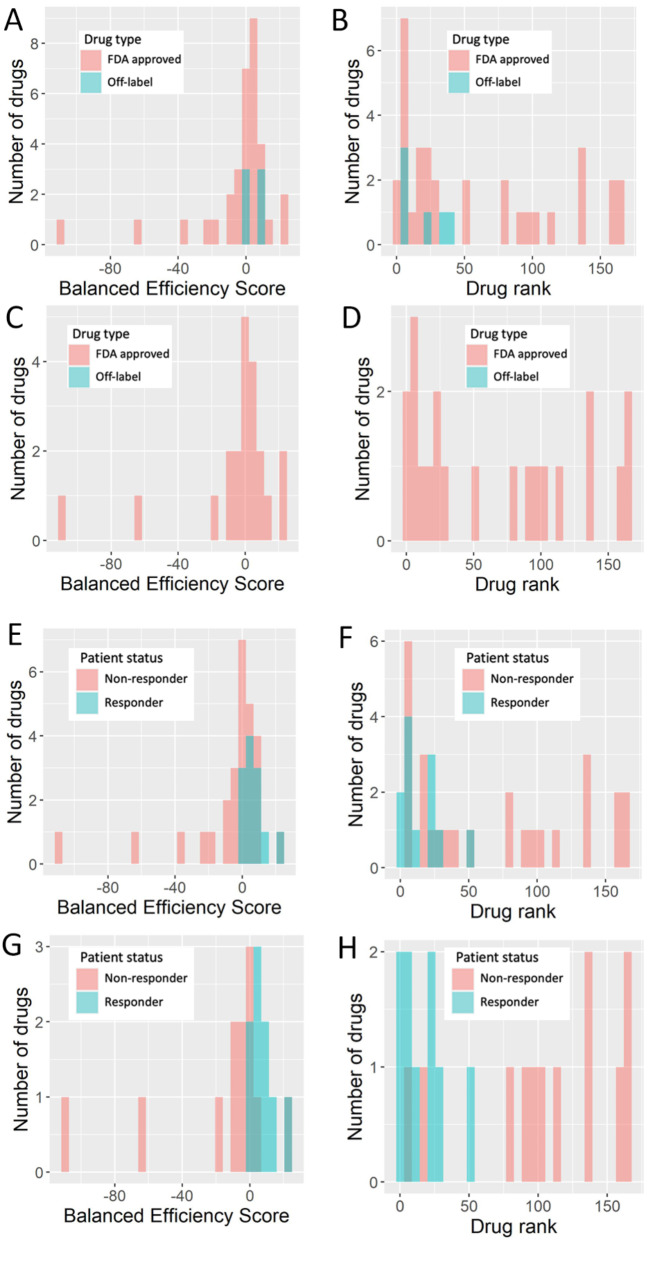
Distribution of BES values and ranks in experimental cohort for all lines of therapy (**ae**, n = 30; **bf**, n = 30) and for first lines of therapy (**cg**, n = 12; **dh**, n = 12). Drug ranks were BES ranks among all drugs in a given patient. Color in **a-d** denotes drugs approved for CRC and experimental therapeutics. Color in **a-d** denotes drugs approved for CRC and experimental therapeutics. Color in **e-h** denotes treatment response. *Responders* group included patients with partial response and stable disease, *non-responders* group included progressive disease patients

By comparing tumor versus normal samples, the output modeled drug efficiency value termed *balanced efficiency score* (BES) was calculated for 159 targeted therapeutics present in the Oncobox database [74]. This allowed to individually rank the drugs according to their BES values. The higher BES suggests higher predicted efficacy of a targeted drug for an individual tumor; overall, positive values suggest potential usefulness of a drug to the patient, whereas zero or negative values predict lack of benefit for the patient treatment [40]. Thus, rank of BES score for a given drug among other drugs investigated in an individual patient can serve as a measure of predicted efficacy for this drug compared to the others [40]. Distributions of BES for drugs investigated here and of their ranks are shown for all lines of therapy (Fig. 4AB) and separately for the first line of therapy (Fig. 4CD).

Both standardly approved and off-label drugs used as the experimental therapy were present in our experimental group (Table 2, Supplementary Table S1). Only standardly approved drugs were included in the first line treatments, whereas off-label drugs were prescribed in the further lines. Interestingly, off-label therapeutics formed a small group of treatments with relatively high BES values and ranks (Fig. 4AB).

We then visualized BES values and ranks distributions in relation to the patient response status for all lines of therapy (Fig. 4EF) and separately for the first line of therapy (Fig. 4GH). In both cases drugs prescribed for the responders had relatively higher BES scores (Fig. 4EG, respectively) and ranks (Fig. 4FH, respectively).

We then statistically compared ranks of Oncobox BES values with the registered clinical outcomes in the experimental patient cohort (Supplementary Table S1). To this end we used the *area under the ROC curve* (ROC AUC) metric that is broadly applicable for assessing biomarker quality in oncology [75–80]. The AUC value is the overall characteristic of biomarker robustness that positively correlates with the quality of a biomarker and varies depending on its sensitivity and specificity in a range between 0.5 and 1 [81]. The standard biomarker quality threshold is 0.7 and greater AUC values indicate good-quality biomarkers, and vice versa [82].

For the available 39 lines of therapy in the experimental cohort (Supplementary Table S1) we compared Oncobox drug ranks versus treatment outcome records (classified as either *control over disease* (positive outcome) or *progressive disease* (negative outcome) (Fig. 4E-H). We found that the Oncobox BES rank as the biomarker could effectively predict CRC response on treatment by targeted therapeutics with AUC = 0.77 (Supplementary Figure S2A). For the first-line only targeted therapy data, there were positive outcomes for 9 patients (6 stable disease, 3 partial responses), and the negative outcomes for 12 patients (progressive disease). AUC-based performance of Oncobox drug score ranks in this case was higher than for the analysis of all available outcomes (AUC = 0.91; Supplementary figure S2B). We additionally tested BES predictive capacity in bevacizumab-only subset of the experimental data. BES predicted bevacizumab response with AUC 0.82 in the first-line therapy group (9 responders, 6 non-responders), and AUC 0.64 in non-first-line therapy group (1 responder, 7 non-responders). This suggests that using biopsy specimens obtained recently before RNA sequencing and BES analysis can result in more informative reports compared to using older biosamples where further lines of therapy were ongoing, because every additional therapy can reshape tumor gene expression profile. Indeed, BES AUC was only 0.64 for combined non-first line treatments.

We then investigated in the same way performance of the Oncobox drug ranking algorithm for the previously published literature datasets of CRC expression profiles annotated with the targeted drug treatment outcomes. We identified clinical response-annotated datasets GSE19860 and GSE19862 with Affymetrix Human Genome U133 Plus 2.0 microarray gene expression profiles for 12 and 14 CRC patients, respectively, who were treated with bevacizumab. Patients from dataset GSE19860 were treated by the combination therapy of bevacizumab plus mFOLFOX6, whereas patients from dataset GSE19862 received bevacizumab therapy.

**Fig. 5:**
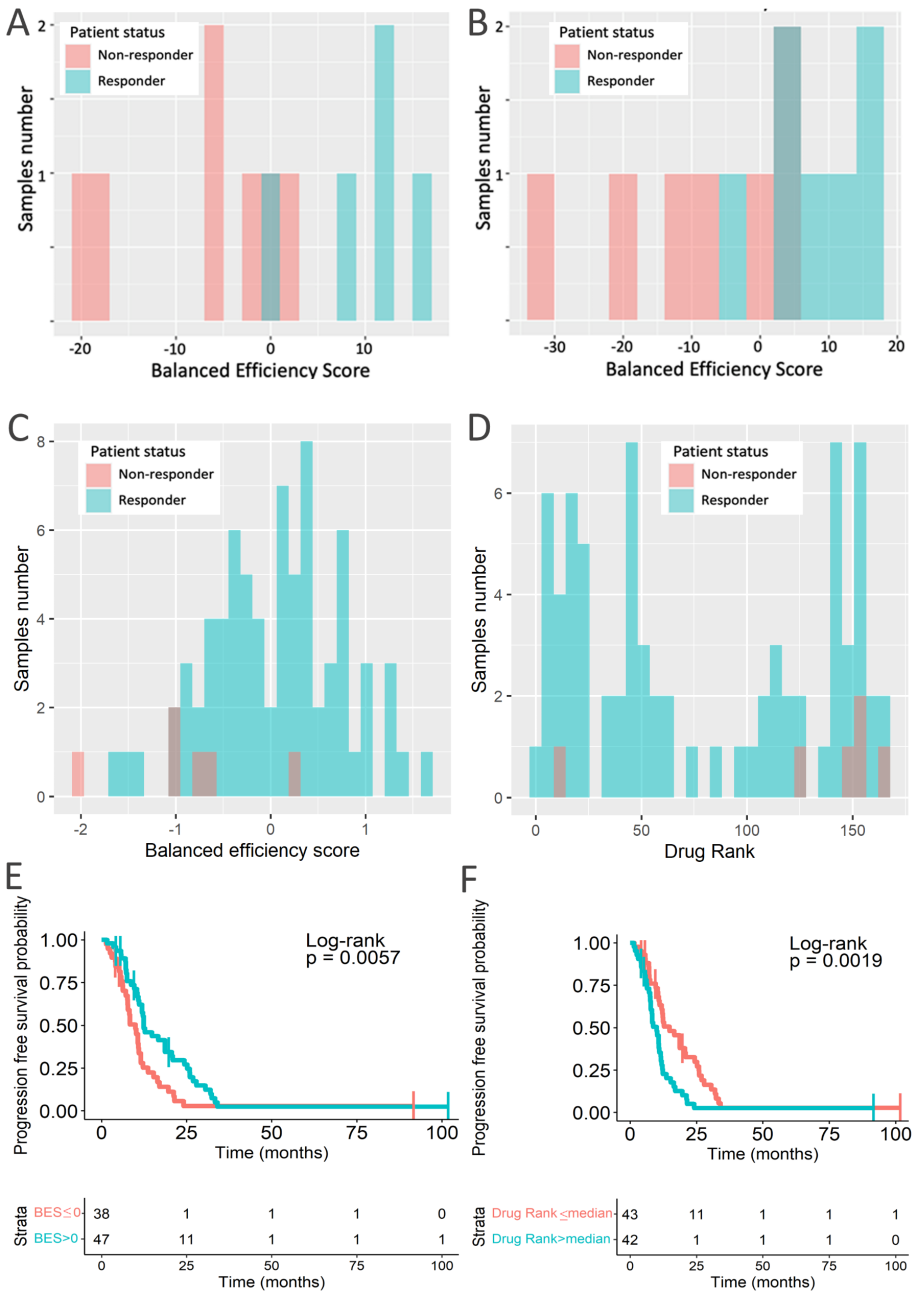
BES performance for the literature CRC gene expression profiles. **(a)** GSE19860 dataset, AUC = 0.94, 5 *responders* and 7 *non-responders.***(b)** GSE19862 dataset, AUC = 0.90, 7 *responders* and *7 non-responders*. **(c)** GSE104645 dataset, AUC = 0.84, 75 *responders* and 6 *non-responders*. **(d)** GSE104645 dataset by Drug Rank AUC = 0.73, 75 *responders* and 6 *non-responders*. **(ef)** PFS analysis of GSE104645 cohort stratified by BES value **(e)** or rank **(f)**

We calculated bevacizumab BES based on normalized gene signal intensity values [83] and observed its high performance in predicting drug response for those datasets as well (AUC 0.94 and 0.90 for GSE19860 and GSE19862, respectively; Fig. 5AB).

We further validated BES predictive capacity on publicly available gene expression dataset GSE104645. We found that BES value was an effective response predictor for bevacizumab (AUC = 0.84) and was strongly associated with PFS (HR = 0.53, CI 0.33–0.84, log rank test p-value 0.0057, Fig. 5CE). BES rank for bevacizumab showed the same trend (AUC = 0.73) and was strongly associated with PFS (HR = 2.06, CI 1.29–3.29, log rank test p-value 0.0019, Fig. 5DF). However, BES was ineffective for predicting results of second-line treatment with cetuximab (p-value ~ 0.48, data not shown), and in this case showed no association with PFS (p-value ~ 0.89, data not shown), which agrees with our previous results on lower efficacy for non-first line treatments predictions (Supplementary Figure S2).

Alternatively, this can be the result of a lower predictive capacity of BES towards EGFR-targeted monoclonal antibodies, in contrast to bevacizumab.

We then investigated performance of Oncobox drug ranking for another relevant clinically annotated gene expression dataset extracted from the TCGA project database. We found that the Oncobox BES rank in the same settings as for the previous patient cohorts predicted treatment response with AUC = 0.74 (Supplementary Figure S3).

### Comparison of algorithmic drug ranking and experimental progression-free survival data

**Fig. 6:**
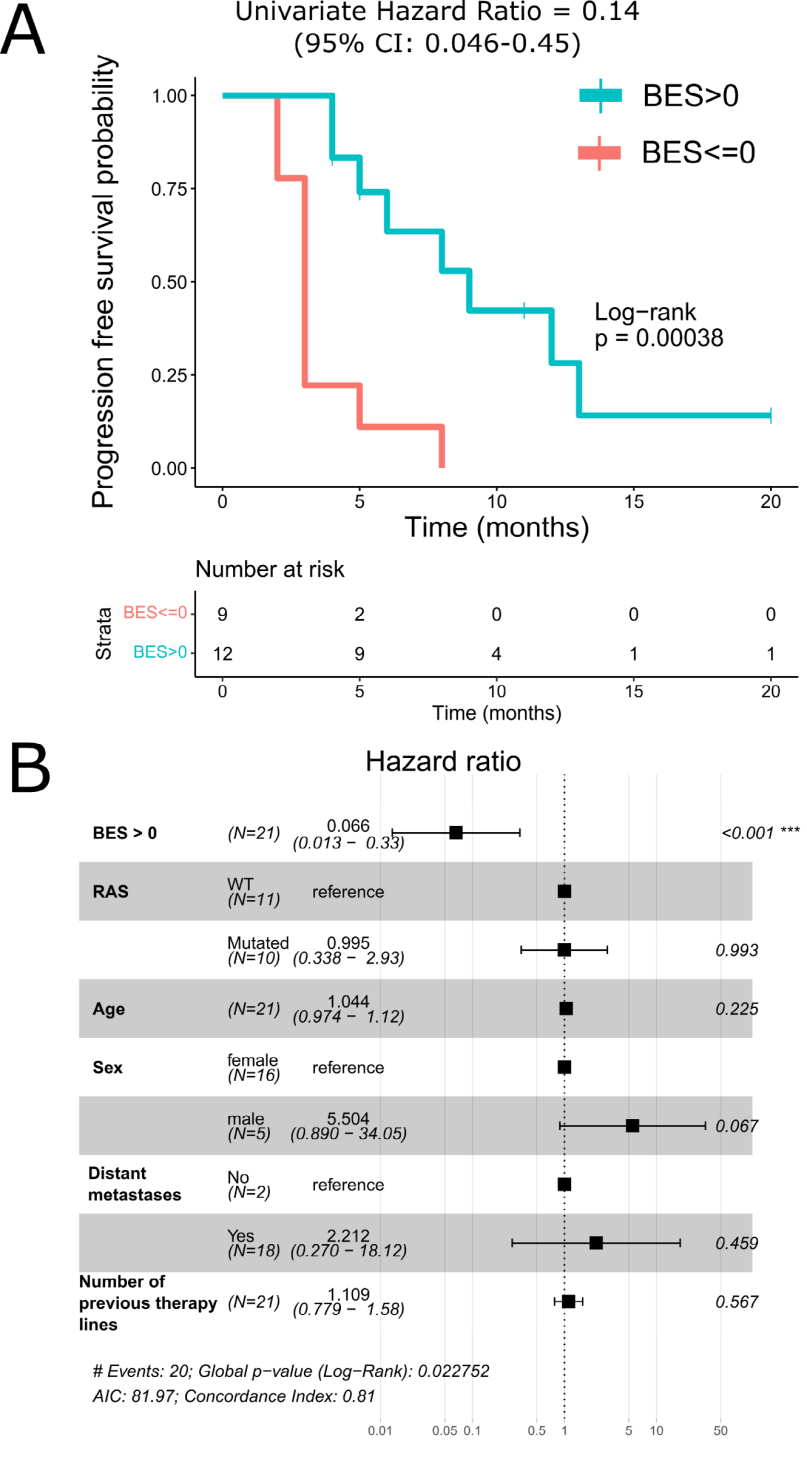
Survival analysis of experimental dataset for association of PFS and drug BES value. **(a)** – Kaplan-Meier plot based on grouping by BES score (n = 21). **(b)** – results of the Cox proportional hazards multivariate analysis (n = 21 lines). AIC- Akaike Information Criterion. Hazard Ratios (black squares) for the features are presented on X-scale (log-transformed), whiskers indicate 95% confidence interval. Right column shows Cox proportional-hazards model p-values for Hazard Ratios

We then assessed the ability of Oncobox drug score ranks to predict progression-free survival (PFS) of CRC patients following treatment with the respective targeted drugs. Second and further treatment lines were prescribed to the same patients as the first line, therefore we included only first-line treatment outcomes into this analysis. Median PFS for the first line therapy data was 5 months in the entire experimental cohort. For the PFS analysis we stratified patients with the available “first line” targeted therapy outcomes in two groups: (*i*) those who received drug with BES > 0 (predicted as effective), or (*ii*) with BES ≤ 0 (predicted as ineffective), summarized on Table 1. The threshold for BES was established in the previous studies [74, 84]. Univariate survival analysis showed the significant difference in PFS between those two cohorts: patients who received in the first line drugs with BES > 0 demonstrated higher PFS (Hazard ratio = 0.14, log-rank *p* = 0.00038), Fig. 6 A.

We then compared the significance of drug score ranks for PFS with the possible impact of patient age, sex, cancer stage and presence of *RAS* family gene mutation using Cox proportional hazards multivariate analysis (Fig. 6B). This type of analysis also confirmed PFS differences between the two cohorts of patients with BES > 0 and BES ≤ 0 (*p* < 0.001). *RAS* family mutation status was insignificant which agrees with the previous report [85] that on the background of adjuvant chemotherapy (as in this study) *RAS* family mutation is not linked with PFS in CRC. Number of previous lines of therapy was not significant in this analysis possibly due to low variability of this parameter between the patients: 9 patients did not receive prior therapy, 6 patients received one line, 3 patients – 2 lines, 1 patient – 3 lines and 2 patients – 5 lines (Table S1).

## Discussion

In this paper we experimentally investigated RNA sequencing gene expression profiles for fourteen CRC patient FFPE tumor biosamples linked with the data on clinical response for 39 lines of therapy containing nine different targeted cancer drugs. Molecular data, such as gene expression and molecular pathway activation levels, is a rich source of information for attempting to personalize cancer drug prescriptions [29, 86, 87]. By using bioinformatic platform Oncobox [34, 36, 39] we modeled here the possible outcomes of nine targeted drugs based on RNA sequencing data and compared the output results with the observed clinical response data.

To this end, the Oncobox drug score (BES) ranks were compared with the therapy response records. We found that the BES ranks could effectively predict tumor response for the experimental CRC patients for the sets of all available therapeutic lines (AUC = 0.77) and for the first lines of therapy after obtaining tumor biopsies (AUC = 0.91). This is in line with the previous literature on the Oncobox method ability to predict gastric cancer outcomes after treatment with ramucirumab, a VEGF receptor-specific targeted therapeutic, also for the FFPE-derived RNA sequencing profiles. In that case AUC was 0.75 for using ramucirumab as the monotherapy, or 0.7 for its use in combinations with other non-targeted chemotherapy drugs [88]. The performance of drug score ranks for the first line of therapy was significantly higher than for all lines of therapy, thus suggesting that using more recent biopsy specimens is highly influential for the quality of drug treatment response predictions using RNA sequencing data.

Metastatic lesions may have strongly different molecular features than the primary tumors. Liu et al. analyzed matched primary and metastatic CRC samples and did not find significant differences in oncogenic mutations and copy number variations, however identified gene networks associated with CRC metastasis, which may have an impact on treatment outcomes [89]. To further investigate this issue, we performed an additional analysis and excluded metastatic tumors from our experimental dataset (three out of twelve samples). In this case, we obtained AUC 0.7 for all therapy lines analysis, 0.92 for the first-line therapy only, and 0.83 for bevacizumab as the first-line therapy. These results did not differ significantly from the analysis of the entire experimental dataset.

Pentheroudakis et al. previously aimed at identification of a gene expression signature associated with progression-free survival after bevacizumab treatment in CRC. The authors developed potentially effective two-gene signatures, which however were not validated independently [90]. We also performed progression free survival (PFS) analysis for the experimental patients to assess the ability of algorithmic drug score to predict PFS in CRC patients. It was found a significant PFS predictor in both univariate (Hazard ratio = 0.14, log-rank *p* = 0.00038) and multivariate (Hazard ratio = 0.066, log-rank *p* < 0.001) survival analysis: patients who received drug with BES > 0 showed higher PFS. However, the current experimental sampling was limited, and further clinical investigations are needed to provide sufficient line of evidence for introducing algorithmic RNA-based drug scoring to the clinical routine.

The results obtained are in agreement with the hypotheses (*i*) that RNA sequencing data can help personalizing cancer drug prescriptions, and (*ii*) that not only the fresh or freshly-frozen specimens can be used for the analysis, whereas the FFPE-derived materials can be also informative. The latter is important in terms of feasibility of storing tumor biosamples, as FFPE tissue blocks can be stored at room temperature for years, still serving as the source of RNA of acceptable quality for gene expression studies [33]. While RNA is usually degraded in FFPE biosamples, RNAseq still can be used to produce gene expression profiles that give a highly correlated figure with the profiles obtained for the respective fresh tissues biosamples, e.g. as shown by Ben-Moshe et al. [89].

The method of algorithmic drug scoring completely relies on the gene profiling data; thus, RNA sequencing procedure should be standardized for obtaining reproducible results. Another limitation is that due to its rationale it can only rank cancer drugs with characterized molecular specificities (called targeted drugs), whereas many useful non-targeted chemotherapeutics remain outside the analysis.

When comparing treatment responders versus non-responders we also found five molecular pathways that can be indicative of good or poor CRC treatment outcome (Supplementary Table S4).

The most strongly activated pathway connected with poor prognosis was the *KEGG Bladder cancer Main Pathway* and the most strongly activated pathway linked with the favorable prognosis was the *NCI p73 transcription factor network Pathway apoptosis and DNA repair*. The latter was activated in the favorable prognosis group and had several upregulated tumor suppressor genes promoting DNA repair and apoptosis [91]: BUB1/BUB3, BRCA2/PALB2, MCM8, CIDEA, WWOX, EP300, PLK3, CDK5 and PRKCQ (Fig. 3). It had upregulated VEGF (target of bevacizumab, Table 3) which indicates that favorable prognosis is connected with activation of molecular signaling that can be targeted by bevacizumab. This is also in line with previous study by Kotulak et al., where the authors revealed decreased expression of p73 in colorectal cancer [92]. Moreover, Uboveja et al. showed that p73 plays a critical role in suppression of colon cancer metastasis [93], which in turn may be associated with prognosis. On the contrary, *KEGG Bladder cancer Main Pathway*, which was activated in the poor prognosis group, had no molecular targets of any drug explored in this study. Thus, contrast activation features of these pathways in CRCs may be important for drug response. However, this observation needs to be validated in further independent research using cohorts with a more controlled set of patients with less diverse tumor characteristics e.g. focused on particular molecular subtype as described previously [97].

Taken together, our results support the hypothesis that utilizing targeted therapies in CRC can be improved by using RNA sequencing based algorithmic analysis. However, current study has certain limitations: first, different types of colon and rectal cancers were analyzed altogether. Second, nine targeted drugs were prescribed to the patients in experimental cohort. In future studies, separate analyses for individual drugs and specific cancer subtypes should be conducted to evaluate predictive values of gene expression profiling for prescribing therapy in colorectal cancer.

## Conclusion

In this study we analyzed experimental and publicly available gene expression profiles with linked targeted drug treatment outcomes. Oncobox bioinformatical platform was used to simulate targeted drug efficiencies in individual patients. Oncobox demonstrated high predictive capacity, especially when freshly obtained biopsies were used to predict sensitivity to bevacizumab. Our results suggest that gene expression profiling of tumor FFPE materials may be helpful for personalizing prescriptions of targeted therapeutics in CRC.

## Electronic supplementary material

Below is the link to the electronic supplementary material.

Supplementary Material 1

Supplementary Material 2

Supplementary Material 3

Supplementary Material 4

Supplementary Material 5

Supplementary Material 6

Supplementary Material 7

Supplementary Material 8

## Data Availability

Supplemental material for this article is available online. The original sequencing data were deposited to NCBI SRA with accession number PRJNA663280 allowing a free access: https://www.ncbi.nlm.nih.gov/bioproject/PRJNA663280/.
