# Supplementary figures and images for: Personalized targeted therapy prescription in colorectal cancer using algorithmic analysis of RNA sequencing data
Source: BMC Cancer. 2022 Oct 31;22:1113. doi: 10.1186/s12885-022-10177-3 (PMC9623986; doi:10.1186/s12885-022-10177-3)

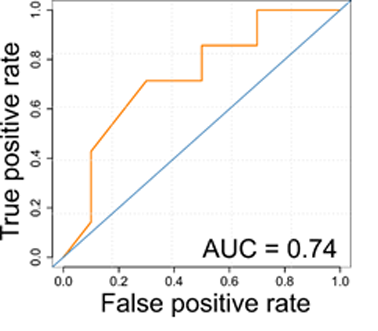

Supplement: Supplementary file 3 — Supplementary Material 3 [file 12885_2022_10177_MOESM3_ESM.png]

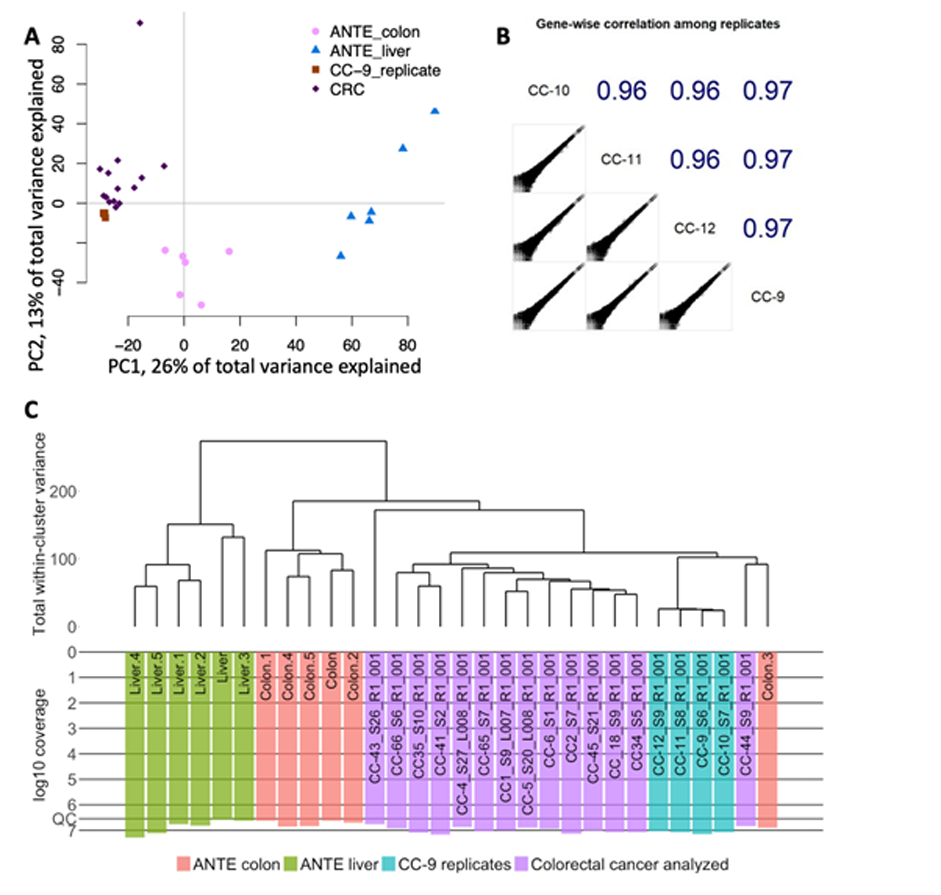

Supplement: Supplementary file 5 — Supplementary Material 5 [file 12885_2022_10177_MOESM5_ESM.png]

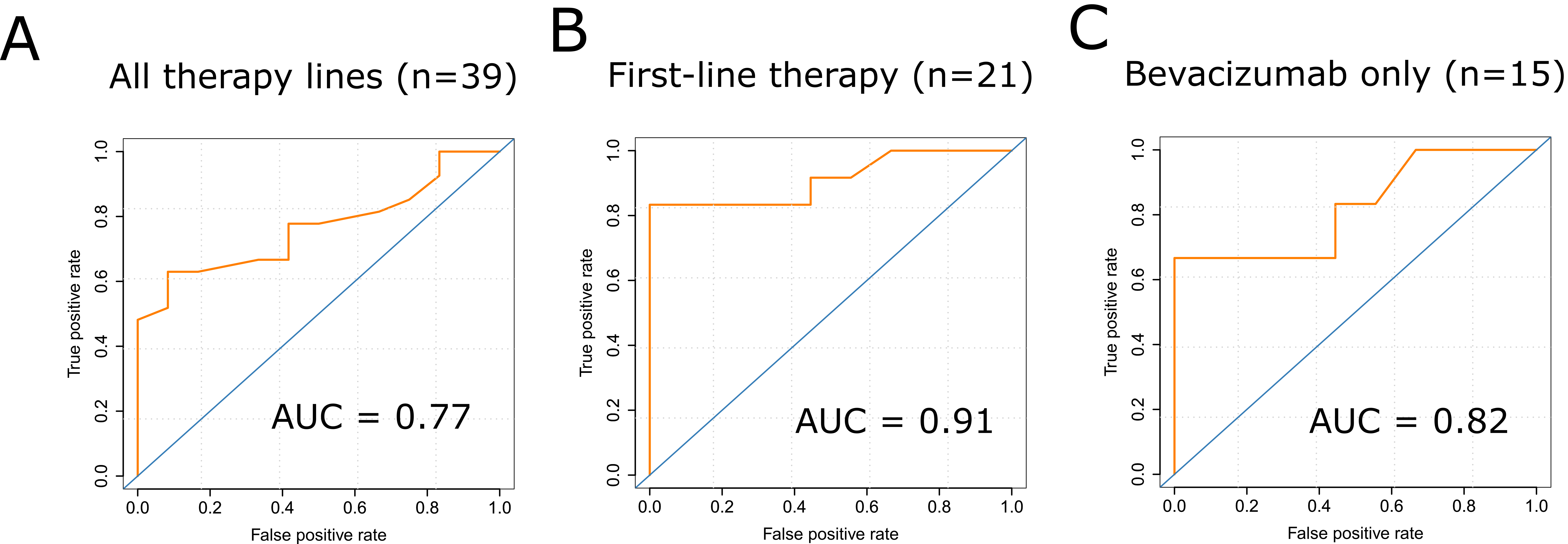

Supplement: Supplementary file 6 — Supplementary Material 6 [file 12885_2022_10177_MOESM6_ESM.png]
