# Supplementary material for: Personalized targeted therapy prescription in colorectal cancer using algorithmic analysis of RNA sequencing data
Source: BMC Cancer. 2022 Oct 31;22:1113. doi: 10.1186/s12885-022-10177-3 (PMC9623986; doi:10.1186/s12885-022-10177-3)
Supplement: Supplementary file 8 — Supplementary Material 8 [file 12885_2022_10177_MOESM8_ESM.docx]

**Supplementary Materials**

Supplementary Table S1. Patient clinical records.

Supplementary Table S2. BES for replicates (rank and absolute values with standard deviation).

Supplementary Table S3. Association of gene expression levels and PFS in the experimental, Syn2623706 and TCGA datasets..

Supplementary Table S4. Association of pathway activation levels and PFS in the experimental, Syn2623706 and TCGA datasets.

Supplementary Figure S1. Clustering and data quality assessment of experimental RNA sequencing profiles. All analyses were performed in the space of log10-transformed DESeq2-normalyzed read counts. (a) - Principal Component Analysis of 14 samples of interest (CRC), four replicates of sample CC-9, six samples of normal colon and six samples of normal liver from ANTE database. (b) – correlation plot between four technical replicates of CC-9 sample. Bottom left side shows gene-wise correlations among replicates, top right side shows Person correlation coefficients. (c) – hierarchical clustering of experimental CRC samples, CC-9 sample replicates, normal colon and normal liver samples. Dendrogram was built using ward.d2 method and Euclidean distance. Bar height shows log10-transformed number of sequencing reads uniquely mapped on genes.

Supplementary Figure S2. ROC curves for comparison of overall response rate and drug BES rank. (a) – all lines of therapy (30 lines, 14 patients). (b) – first lines of therapy (12 lines, 12 patients). (c) analysis limited to bevacizumab only treatment outcomes

Supplementary Figure S3. ROC curve for comparison of overall response rate and drug BES rank in TCGA data (17 colon cancer patients annotated with drug response data).
